# Biological Role of Pazopanib and Sunitinib Aldehyde Derivatives in Drug-Induced Liver Injury

**DOI:** 10.3390/metabo12090852

**Published:** 2022-09-11

**Authors:** Maud Maillard, Cécile Arellano, Christelle Vachoux, Christine Chevreau, Nicolas J. Cabaton, Frédéric Pont, Nathalie Saint-Laurent, Thierry Lafont, Etienne Chatelut, Fabienne Thomas

**Affiliations:** 1 Centre de Recherches en Cancérologie de Toulouse, Université de Toulouse, Inserm, Université Toulouse III-Paul Sabatier, 2 Avenue Hubert Curien, CS53717, CEDEX 1, 31037 Toulouse, France; 2 Institut Claudius Regaud IUCT-Oncopole, CEDEX 9, 31059 Toulouse, France; 3 Toxalim (Research Centre in Food Toxicology), Université de Toulouse, INRAE, ENVT, INP-Purpan, UPS, 31027 Toulouse, France

**Keywords:** aldehyde, hepatotoxicity, pazopanib, reactive metabolites, sunitinib

## Abstract

Tyrosine kinase inhibitors pazopanib and sunitinib are both used to treat advanced renal cell carcinoma but expose patients to an increased risk of hepatotoxicity. We have previously identified two aldehyde derivatives for pazopanib and sunitinib (P-CHO and S-CHO, respectively) in liver microsomes. In this study, we aimed to decipher their role in hepatotoxicity by treating HepG2 and HepaRG hepatic cell lines with these derivatives and evaluating cell viability, mitochondrial dysfunction, and oxidative stress accumulation. Additionally, plasma concentrations of P-CHO were assessed in a cohort of patients treated with pazopanib. Results showed that S-CHO slightly decreased the viability of HepG2, but to a lesser extent than sunitinib, and affected the maximal respiratory capacity of the mitochondrial chain. P-CHO decreased viability and ATP production in HepG2. Traces of P-CHO were detected in the plasma of patients treated with pazopanib. Overall, these results showed that P-CHO and S-CHO affect hepatocyte integrity and could be involved in the pazopanib and sunitinib hepatotoxicity.

## 1. Introduction

Drug-induced liver injuries (DILIs) are the primary cause of acute liver failure and drug withdrawal in the United States, where they represent a major clinical and economic concern for healthcare [1]. Pazopanib (Votrient^®^) and sunitinib (Sutent^®^) are two oral tyrosine kinase inhibitors (TKIs) used to treat metastatic renal cell carcinoma (mRCC). Both drugs are “black-boxed” by the U.S. Federal Drug Administration (FDA) for an increased risk of acute hepatotoxicity [2,3]. The mechanisms of TKI-induced liver toxicity are not fully understood, but recent works have highlighted its multifactorial etiology; particular interest has been shown in the formation of reactive metabolites from the biotransformation of TKIs such as lapatinib [4], famitinib [5], pazopanib, sunitinib [6], and more recently, olmutinib [7]. Their toxic potential is thought to be caused by their propensity to generate electrophilic radicals or moieties (such as aldehydes or intermediate semi-quinone radicals leading to reactive quinone imines) that can covalently bind intracellular components, affect cell structure, and induce cell death. These structural alert patterns may result from the biotransformation of the parent molecule by phase I enzymes such as cytochromes P450 (CYP450), making the liver the privileged place for their production [8].

Alteration of the structure and/or function of mitochondria is supposed to cause DILIs [9]. This hypothesis has been reported for ponatinib, regorafenib, sorafenib [10], sunitinib [11,12], and lapatinib [4] in the hepatocarcinoma cell line HepG2 and for crizotinib, dasatinib, ponatinib, regorafenib, and sorafenib in human HepaRG cells [13]. However, except for lapatinib, the involvement of reactive metabolites has not yet been investigated, and studies have only focused on parent molecule-induced toxicity.

Using metalloporphyrin, a chemical catalytic system that mimics specific CYP450-type oxidation, our team has previously identified two aldehyde derivatives for pazopanib and sunitinib [14]. Their reactivity was shown by the in vitro detection of covalent adducts to lysine in liver microsomes and recombinant CYP3A4 incubations.

In this study, we question the putative role of pazopanib and sunitinib aldehyde derivatives in their clinical hepatotoxicity. To this end, we evaluated the ability of these aldehydes to impair hepatocyte viability by focusing on the mitochondrial function and the accumulation of intracellular oxidative stress. Finally, we attempted to evaluate the relationship between plasma concentrations of pazopanib aldehyde and clinical hepatic side effects reported for patients treated with pazopanib for mRCC.

## 2. Materials and Methods

### 2.1. Chemicals and Solvents

Sunitinib L-malate and pazopanib hydrochloride (HCl) were purchased from Alsachim (Illkirch Graffenstaden, France, purity > 98%). Aldehyde metabolites of pazopanib (P-CHO) and sunitinib (S-CHO) were synthesized by chemical synthesis (Institut de Chimie et Biochimie Moléculaires et Supramoléculaires ICBMS, Université Claude Bernard, Villeurbanne, France). Structures were confirmed by NMR and LC-MS analysis. Stock solutions were prepared in dimethyl sulfoxide (DMSO, Sigma-Aldrich, St. Louis, MO, USA) and stored at −20 °C. Pazopanib HCl was dissolved in dimethylformamide (DMF) for UPLC-MS/MS analysis. L-buthionine sulfoximine (BSO), sodium cyanoborohydride (NaBH_3_CN), and ammonium formate were purchased from Sigma-Aldrich; formic acid was provided by Merck (Darmstadt, Germany). Acetonitrile and methanol were provided by Biosolve (Valkenswaard, The Netherlands, UPLC-MS/MS grade) and isopropanol was purchased from Merck. Blank plasma from healthy donors was provided by the French National Blood Service (EFS, Toulouse, France).

### 2.2. Cell Culture

Two hepatic cell lines were used to evaluate the cytotoxicity of P-CHO and S-CHO. The human hepatocarcinoma cell line HepG2 (ATCC reference HB-8065) was maintained in complete high-glucose media (Dulbecco’s modified Eagle Medium, DMEM, glucose 4.5 g/L, Sigma) supplemented with 10% (*v*/*v*) of fetal bovine serum (FBS), 0.1% of antimycotic solution (Plasmocin™ prophylactic, InvivoGen, Toulouse, France), and 1% of an antibiotic cocktail (penicillin and streptomycin, Thermo Fisher Scientific, Waltham, MA USA). For culture in glucose-depleted conditions, the medium was replaced with complete medium supplemented with 10 mM galactose, 2 mM L-glutamine, antibiotic-antimycotic solution, 5 mM HEPES, and 1 mM sodium pyruvate.

The cytotoxicity experiments with the HepaRG cell line were performed at the INRAE/Toxalim Institute (Toulouse, France). Before experimental usage, they were seeded for two weeks at a density of 2.6 × 10^4^ cells/cm^2^ in a growth medium composed of Williams’ E medium supplemented with 10% FCS, 100 units/mL penicillin, 100 mg/mL streptomycin, 5 mg/mL insulin, 2 mM glutamine, and 5 × 10^−5^ M hydrocortisone hemisuccinate. After two weeks, cells were shifted to the same culture medium supplemented with 2% DMSO (differentiation medium) for two additional weeks (confluent DMSO-treated cells) [15]. The medium was renewed every two to three days.

### 2.3. LDH Release, Caspase 3/7 Activation and ATP Measurement

Acute cytotoxicity was assessed by measuring the LDH liberation following membrane damage with the CytoTox-ONE™ Homogeneous Membrane Integrity Assay (Promega, Madison, WI, USA). Briefly, HepG2 cells were seeded at 20,000 cells per well and exposed for 24 h to increase the concentrations of components (1–100 µM for sunitinib, S-CHO, and P-CHO and 1–50 µM for pazopanib due to its limited solubility in aqueous media). HepaRG cells were seeded at 50,000 cells per well and were exposed to the molecules 24 h later for 24 h. The negative control was the DMSO maximal level, with Triton^®^ X-100 as the positive control. Depletion of intracellular glutathione was induced by pre-treating cells with 250 µM of BSO 24 h prior to drug administration. Fluorescence (560/590 nm) was recorded using a Clariostar plate reader (BMG Labtech, Ortenberg, Germany) for HepG2 cells and a Tecan Infinite 200 (Lyon, France) for HepaRG cells. Data were integrated with Mars Data Analysis software (v.1.51, BMG Labtech) and iControl V1.30 software. Induced apoptosis was investigated by measuring caspase 3/7 activity with the Caspase-Glo^®^ 3/7 luminescent assay (Promega). Proportional luminescence was recorded using a plate reader. ATP content was assessed in cells cultured in high-glucose or galactose media with the CellTiter-Glo^®^ Luminescent Cell Viability Assay (Promega) after cell lysis.

### 2.4. Evaluation of the Mitochondrial Respiration in HepG2 Cells

Cellular respiration was measured with the mitochondrial oxygen consumption rate (OCR, expressed in pmol O_2_/min) in HepG2 with Seahorse XF Cell Mito Stress for a Seahorse XF24e analyzer (Seahorse Biosciences, North Billerica, MA, USA). Cells were seeded at a concentration of 20,000 cells/well in high-glucose medium and were exposed to aldehydes at non-toxic concentrations (1–40 µM). One hour before the experiment, the medium was replaced by a low-glucose solution (Agilent, Santa-Clara, CA, USA) supplemented with pyruvate 1 mM, glutamine 2 mM, and glucose 10 mM. NaOH 0.1 M was used to adjust the pH to 7.4. The plate was incubated at 37 °C, 0% CO_2_ for one hour. Mitochondrial respiratory chain inhibitors were prepared in that freshly made medium. The assay consisted in measuring basal respiration followed by the sequential addition of an ATP synthase inhibitor, oligomycin (2 µM), followed then by maximal stimulation of the mitochondrial electron transport chain by adding carbonyl cyanide m-chlorophenylhydrazone (CCCP, 2 µM, an uncoupling agent dissipating the proton gradient). Finally, adding rotenone- and antimycin A- (0.5 µM each) inhibited mitochondrial complexes I and III, respectively, and allowed us to determine extramitochondrial respiration. Maximal respiratory capacity was calculated after subtracting extramitochondrial respiration. The assay was conducted over 96 min, and data were normalized to the protein content with the Pierce™ BCA Protein Assay Kit (ThermoFisher, Waltham, MA, USA).

### 2.5. Mitochondrial Superoxide Accumulation in HepG2 Cells

The accumulation of mitochondrial superoxide was assessed in live cells using the selective MitoSOX™ Red Mitochondrial Superoxide Indicator (Invitrogen). To evaluate the role of GSH in aldehyde detoxication, cells were exposed to an increasing range of reactive metabolites (1–50 µM) in either the presence or absence of BSO (250 µM), a specific inhibitor of GSH synthesis. Rotenone (50 µM) was used as a positive control. After treatment, cells were gently washed with PBS and covered with 2.5 µM of MitoSOX reagent diluted in PBS. Fluorescence was recorded after incubation (excitation/emission wavelength 510 nm/580 nm), and data were normalized to the protein concentration.

### 2.6. Superoxide Dismutases 1 and 2 Gene Expression in HepG2 Cells

Expression of superoxide dismutase1 and 2 encoding genes (*SOD1* and *SOD2*, respectively) was measured by quantitative polymerase in chain reactions (qPCR) in HepG2 cells exposed to 20 µM of components for either two or six hours. RNA was extracted with the Qiagen RNEasy mini kit (Qiagen, Hilden, Germany) and reverse transcription was performed with the Biorad iScript™ Reverse Transcription Supermix (Biorad, Hercules, CA, USA). The qPCR reaction mix was prepared with SYBR^®^ Green Master Mix (Biorad), 20 µM of corresponding forward and reverse primers designed with BLAST^®^ (https://blast.ncbi.nlm.nih.gov/Blast.cgi, accessed on 24 March 2022) (*SOD1*, 5′-GCAGGTCCTCACTTTAATCC-3’ and 5′-GCCACACCATCTTTGTCA-3′; *SOD2*, 5′-GGGTTGGCTTGGTTTCAA-3′ and reverse 5′-GTGCTCCCACACATCAATC-3′; reference gene β2-microglobulin [*B2M*], 5′-TGAGTGCTGTCTCCATGTTTG-3′ and 5′-TCTGCTCCCCACCTCTAAGTT-3′) and free-DNase water. Amplification was performed in triplicates and repeated on two or three distinct RNA extractions. Expression levels were calculated with the 2^−∆∆Ct^ method using a DMSO 0.1% sample as the calibrator and considering a PCR efficiency of 100% [16].

### 2.7. Proteomic Analysis

A proteomic analysis was conducted to identify the interaction sites of aldehyde metabolites with human liver microsomal proteins. P-CHO and S-CHO (20 µM) were incubated with liver microsomes (0.25 mg/mL, ref. #452161, Corning, NY, USA; Bedford, UK) for 90 min. Irreversible binding was allowed by reductive amination with NaBH_3_CN. Protein purification was performed by one-dimension polyacrylamide gel electrophoresis (1D-PAGE) with the use of sodium dodecyl sulfate (SDS). The bands were reduced and alkylated by D, L-dithiothreitol DTT (10 mM), and 2-chloroacetamide (55 mM) and were digested with trypsin. Peptides were diluted in a buffer containing 10% ACN and 0.05% trifluoroacetic acid. Samples were analyzed by HPLC coupled to a SCIEX 5600 + TripleTOF mass spectrometer. Peptide identification was performed with an MSFragger database search tool applied to the SwissProt human database coupled to research in the Protein DataBank (PDB; http://www.rcsb.org/pdb/, accessed on 24 March 2022). Additional information about mass spectrometry and data analyses can be found in the Supplementary Material.

### 2.8. Patients and Hepatotoxicity Data

Correlations between plasma concentrations of P-CHO and liver parameters were evaluated in 15 patients treated with pazopanib for mRCC and included in the SUP-R clinical trial ((ClinicalTrials.gov Identifier: NCT02555748)), a French pilot study evaluating sunitinib and pazopanib pharmacokinetic parameters and toxicity. Patients were sampled on the first day of the first treatment cycle before intake (T0), and at two, four, six, and eight hours after intake (T2, T4, T6, T8). This schedule was repeated at T0, T2, and T6 on the 15th day of cycle 1 and cycle 2. Patients’ hepatic biochemical parameters were collected before inclusion and during treatment and were graded according to the Common Terminology Criteria for Adverse Events (CTCAE, v.4.03).

### 2.9. Preparation of Patients’ Samples for Pazopanib Aldehyde Quantification

The procedure to extract P-CHO from plasma was slightly adapted from our previous work [6]. Briefly, 250 µL of plasma were precipitated with 750 µL of acetonitrile and centrifuged at 1500× *g* for 20 min at 4 °C. The supernatant was dried at 37 °C until complete solvent evaporation. The extract was dissolved in a mixture composed of methanol and aqueous mobile phase (75/25%), filtered, and analyzed. For quantification, plasma P-CHO standard solutions (0.49 to 250 ng/mL) were prepared and extracted extemporaneously. The experimentation was validated if at least two out of three quality control (QC) samples were ±15% of the targeted value for medium (40 ng/mL) and high (200 ng/mL) QC and ±20% for low QC (2.5 ng/mL).

### 2.10. UPLC-MS/MS Analysis

Extracted samples were analyzed with a UPLC Waters Acquity system equipped with a Waters TQ-S micro mass spectrometer (triple quadrupole detector) added to an electrospray ionization source (ESI). Components were eluted in a linear gradient with sequential changes in the proportion of aqueous eluent (ammonium formate in ultrapure water, pH = 3.2) and organic eluent (acetonitrile with formic acid 0.1% [*v*/*v*]), as previously described [6]. Data were analyzed with MassLynx software (version 4.2, Waters, Milford, MA, USA). The multiple reaction monitoring (MRM) mode was used for mass spectrometry detection. Selected MRM characteristic transitions were 438.0 > 357.0 (loss of SO_2_NH_2_) for pazopanib, 452.0 > 328.0 (loss of SO_2_NH_2_, CHO and CH_3_) and 452.0 > 343.0 (loss of SO_2_NH_2_ and CHO) for P-CHO. S-CHO was detected with 413.1 > 340.1 (loss of N(CH_2_CH_3_)_2_), 413.1 > 297.1 (loss of N(CH_2_CH_3_)_2_-(CH_2_)_2_-NH-CO), and 413.1 > 268.1 (cleavage of the amide bond and loss of CHO).

### 2.11. Statistical Analyses

All experiments were conducted three times, so the data are given as the mean ± standard deviation (s.d.). A one-way ANOVA followed by Dunnett’s post-test procedure was used for comparison with vehicle control. A two-way ANOVA followed by Bonferroni’s post-test procedure was used to compare multiple conditions. *p* value < 0.05 was considered significant. Statistical analyses were performed with R^®^ version 4.1.0 (R Foundation for Statistical Computing, Vienna, Austria) and plots were created with GraphPad Prism 7.0 (GraphPad Software, La Jolla, CA, USA).

## 3. Results

### 3.1. Evaluation of Pazopanib and Sunitinib Aldehyde Cytotoxicity in Liver Cell Lines

**Figure 1:**
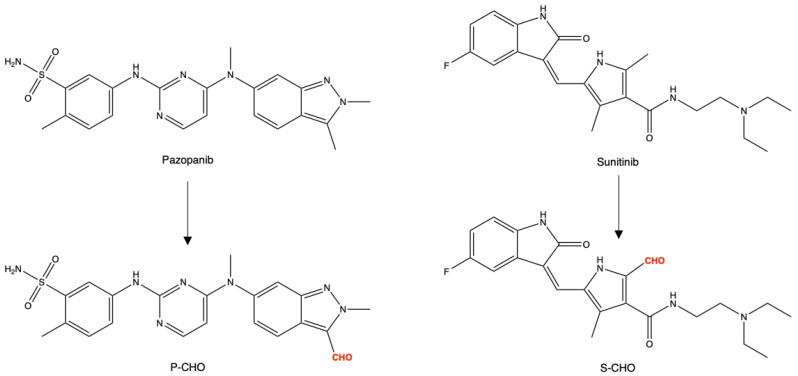
Chemical structures of pazopanib, sunitinib, and aldehyde derivatives (P-CHO and S-CHO, respectively).

The chemical structures of pazopanib, sunitinib, and their derived aldehydes (P-CHO and S-CHO, respectively) are presented in Figure 1.

**Figure 2:**
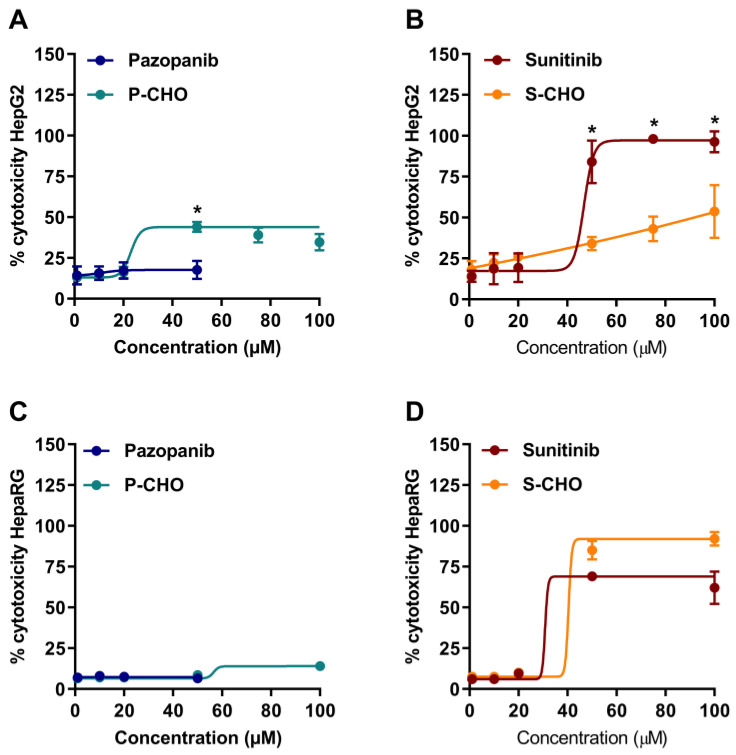
Cytotoxicity on hepatocytes exposed to pazopanib, sunitinib, and their respective aldehydes. Drug cytotoxicity was assessed by measuring lactate dehydrogenase release after 24-h exposure to increasing concentrations of compounds in HepG2 cells (**A**,**B**) and HepaRG cells (**C**,**D**). Results are given as mean and standard deviation (s.d.). * *p* < 0.05 in comparing the parent molecule versus the aldehyde metabolite.

Acute cytotoxicity was tested by exposing HepG2 and HepaRG cells to parent molecules or metabolites for 24 h (1–100 µM, 1–50 µM for pazopanib, due to its limited solubility in aqueous media) and then measuring the release of lactate dehydrogenase (Figure 2). Pazopanib did not affect cell viability at any concentration in both cell lines (Figure 2A,C). In contrast, P-CHO started to be slightly cytotoxic at a concentration of 50 µM with a significant difference at this level compared to pazopanib. Sunitinib and S-CHO both induced cell lysis at 50 µM, with sunitinib having a greater toxic effect (*p* < 0.01) (Figure 2B) in HepG2 cells. As the cytotoxicity of S-CHO was confirmed with the HepaRG cell line (Figure 2D), the rest of the experiments were performed with the HepG2 cell line.

**Figure 3:**
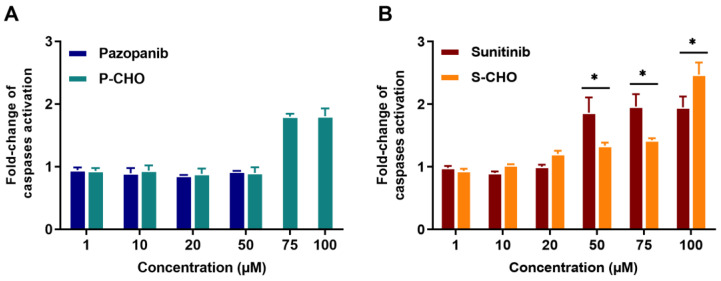
Activation of apoptosis in HepG2 cells exposed to pazopanib, sunitinib, and their respective aldehydes. Apoptosis was evaluated in HepG2 cells by measuring caspases 3 and 7 activity in the presence of pazopanib or P-CHO (**A**) and sunitinib or S-CHO (**B**). Results are given as mean and standard deviation (s.d.) of fold-change of caspases activation compared to control. * *p* < 0.05 in comparing the parent molecule versus the aldehyde metabolite.

The activation of apoptosis was evaluated by measuring the activity of caspases 3 and 7. Apoptosis was increased two-fold when cells were exposed to high concentrations of P-CHO (Figure 3A), and both sunitinib and S-CHO activated apoptosis in a concentration-dependent way (Figure 3B).

### 3.2. Evaluation of the Effect of Pazopanib and Sunitinib Aldehydes on Mitochondrial Function in the HepG2 Cell Line

**Figure 4:**
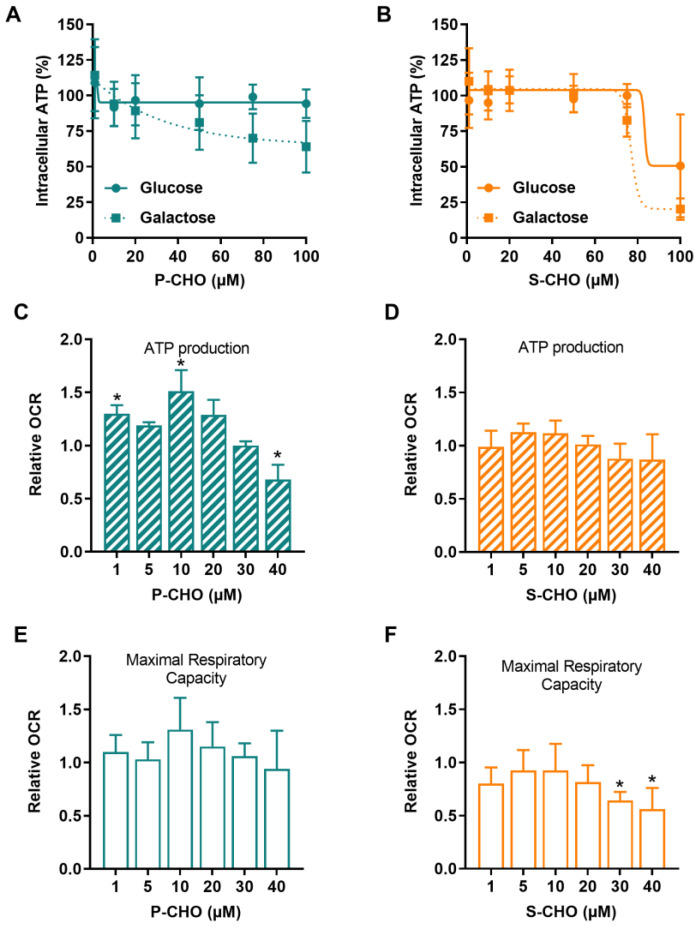
Pazopanib and sunitinib aldehydes impair mitochondrial function. Mitochondrial function was assessed in HepG2 cells by detecting intracellular ATP in cells cultured in a glucose (solid line) or a galactose (dotted line) medium and exposed to increasing concentrations of P-CHO (**A**) or S-CHO (**B**). Relative oxygen consumption rate (OCR) was measured by Seahorse assay in HepG2 cells cultured in a glucose medium. ATP production and Maximal Respiratory Capacity were evaluated for P-CHO (**C**,**E**) and S-CHO (**D**,**F**) and were expressed as the fold-change of OCR measured in non-treated cells. * *p* value < 0.05 compared to non-treated cells.

HepG2 cells were conditioned in galactose complete media to switch cellular ATP production from glycolysis to mitochondrial oxidative phosphorylation. As shown in Figure 4A, P-CHO induced a concentration-dependent decrease in ATP starting at 20 µM, especially in HepG2 cells cultured in galactose. No difference, however, was observed in the glucose culture. S-CHO 100 µM depleted 80% of the ATP content of HepG2 cells cultured in galactose (Figure 4B), whereas it induced lysis in only 50% of cells (Figure 2B).

Mitochondrial respiration was assessed by using a Seahorse XF24e analyzer to measure the oxygen consumption rate (OCR) of HepG2 cells exposed to aldehydes. P-CHO and S-CHO effects on respiratory capacity were tested at concentrations that did not affect cell viability (1–40 µM). As depicted in Figure 4C,E, P-CHO initially increased the mitochondrial respiratory function, and a slight but significant decrease was observed from 10 µM to 40 µM, especially for ATP production. S-CHO did not affect ATP production (Figure 4D), but a significant decrease in maximal respiratory capacity was observed at the highest concentrations tested (30 and 40 µM) (Figure 4F).

### 3.3. Evaluation of Oxidative Stress in the Presence of Reactive Metabolites

**Figure 5:**
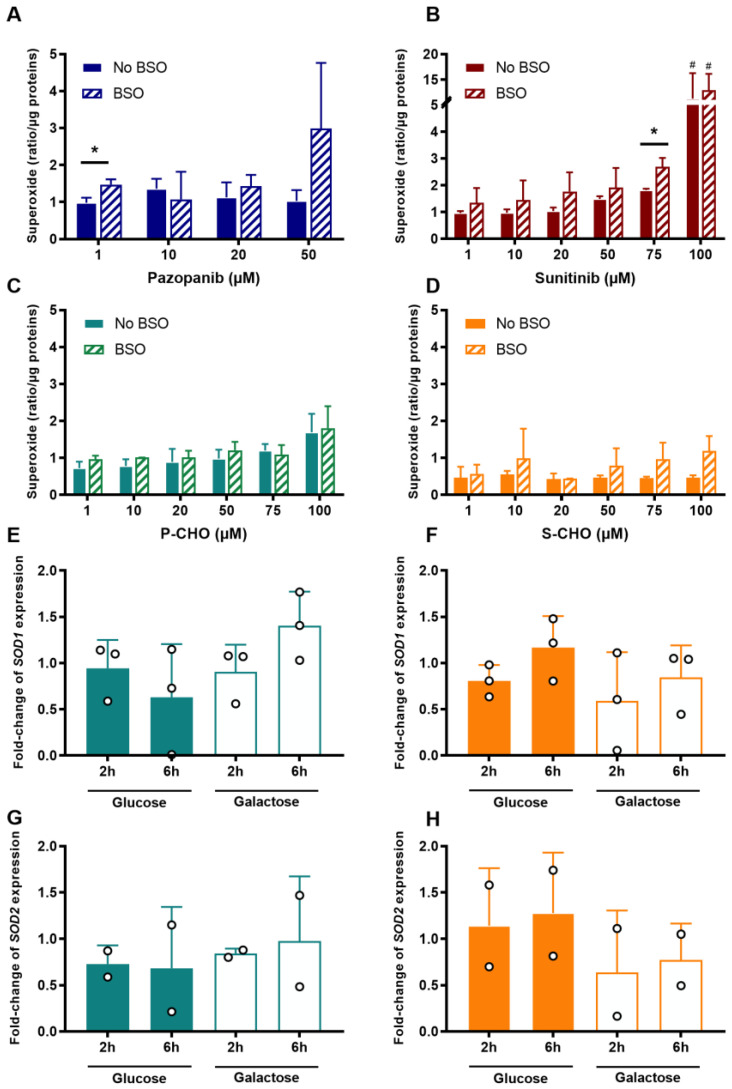
Production of mitochondrial superoxide and superoxide dismutase expression (SOD) in response to the accumulation of oxidative stress in HepG2 cells. Mitochondrial superoxide was measured after exposure to increasing concentrations of pazopanib (**A**), sunitinib (**B**), P-CHO (**C**), or S-CHO (**D**) with or without BSO that depleted intracellular glutathione. Response to the oxidative stress accumulation after 20 µM of P-CHO and S-CHO was evaluated by means of the relative gene expression of *SOD1* (**E**,**F**, respectively) and *SOD2* (**G**,**H**, respectively) measured in HepG2 cells cultured in either glucose or galactose media and incubated for either two or six hours. Expression levels are expressed as a fold-change compared with controls and results from two or three independent experiments (mean ± s.d.). * *p* value < 0.05 for BSO vs. non-BSO comparison. ^#^ *p* value < 0.05 for comparison with non-treated cells.

To investigate the propensity of P-CHO and S-CHO to induce oxidative stress, cells were cultured both with and without BSO, which is known to reduce glutathione cellular levels by inhibiting γ-glutamylcysteine synthetase. In Figure 5, mitochondrial superoxide was detected by means of the MitoSOX indicator in live HepG2 cells and compared with superoxide dismutase (SOD) expression in response to oxidative stress.

The increase in mitochondrial superoxide was marked in HepG2 cells exposed to a high concentration of pazopanib in the presence of BSO (i.e., 50 µM in the absence of glutathione, Figure 5A). In contrast, it only slightly accumulated in a concentration-dependent manner in cells exposed to P-CHO, with no difference between the presence or absence of BSO except at high concentrations (Figure 5B). S-CHO treatment did not result in an accumulation of reactive mitochondrial species under normal conditions, contrary to sunitinib, especially at its highest concentration (Figure 5C,D). Glutathione depletion favored superoxide accumulation in the presence of aldehydes without increasing their cytotoxicity (see Supplementary Figure S1).

To evaluate the response to possible superoxide production, *SOD1* and *SOD2* expression was measured by quantitative RT-PCR in HepG2 cells exposed to 20 µM of P-CHO or S-CHO in galactose (i.e., mitochondrial production of ATP) and high-glucose (i.e., ATP production from glycolysis) media for either two or six hours. Results showed that neither expression of *SOD1* nor *SOD2* was affected in the presence of P-CHO (Figure 5E,F) or S-CHO (Figure 5G,H), except for a slight increase in fold-change mRNA for *SOD1* in cells exposed to P-CHO for six hours in a galactose medium (Figure 5E, non-significant result).

### 3.4. Proteomic Analysis of Aldehyde Binding to Liver Microsomes Proteins

**Figure 6:**
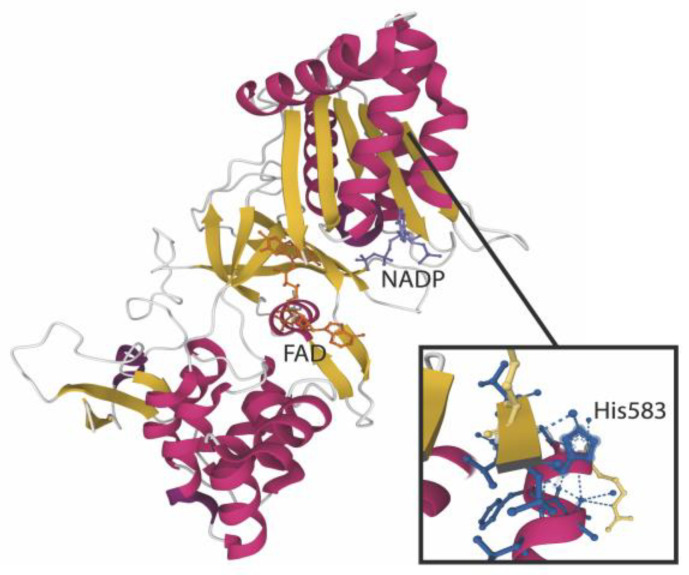
Pazopanib aldehyde interaction with NADPH-cytochrome P450 oxidoreductase. The structure of the POR enzyme (PDB reference: 3QFS [17]) is depicted here with the major cofactors NADP and FAD. The enlarged view focuses on His583 modified by P-CHO after incubation with microsomes.

P-CHO and S-CHO adducts to lysin residues were previously identified in a microsomal fraction of the human liver [6]. Given that protein adducts can contribute to reactive metabolites toxic effects, we attempted to identify the microsomal proteins targeted by our aldehydes by high-performance liquid chromatography coupled with mass spectrometry (HPLC-MS/MS). The total ion chromatogram of the digested microsomes incubated with P-CHO is shown in Supplementary Figure S2. A peptide belonging to the CYP450 coenzyme NADPH-cytochrome P450 reductase was shown to be modified by P-CHO in the histidine located at position 583 (Figure 6 and Supplementary Table S1). The fragmentation MS/MS spectrum can be found in Supplementary Figure S3.

### 3.5. Detection and Origin of Aldehyde Metabolites In Vivo

To determine if the clinical hepatotoxicity of pazopanib could be related to the production of its aldehyde derivative, we measured the plasma concentrations of P-CHO in 15 patients included in the SUP-R clinical trial.

Traces of P-CHO were detected (mean concentration = 6.45 ng/mL, s.d. = 4.30 ng/mL) in the plasma with concentrations showing similar kinetic profiles compared to pazopanib. The median metabolite: parent molecule ratio was 0.026% ([min = 0.009%; max = 1.924%], and Spearman’s rank correlation was ρ = 0.75, *p* < 0.001).

In the cohort, five patients had at least one abnormality of liver biochemistry (all grades and all cycles included). P-CHO mean residual concentration at day 15 of the first cycle was 9.16 ng/mL (±3.65 ng/mL), not significantly different from that of patients with no liver abnormality (mean concentration = 7.21 ng/mL (±3.35 ng/mL), Wilcoxon’s signed-rank *p* = 0.639). S-CHO could not be quantified in patients’ plasma because of analytical issues. Notably, we attempted to decipher the origin of P-CHO and S-CHO by incubating pazopanib with microsomes in the presence of specific inhibitors of CYP450 and with cytosol with inhibitors of alcohol dehydrogenase, as aldehydes may result from alcohol oxidation. All methods and results are detailed in the Supplementary section. Only small amounts of aldehyde were detected, and the signal was at its highest level in inactive microsomes, meaning that the contribution of hepatic enzymes in their formation is probably minor (Supplementary Figure S4).

Moreover, we observed residual signals of P-CHO and S-CHO in microsomal and cytosolic incubations, thus we sought to detect these aldehydes in plasma spiked with pharmaceutical tablets of pazopanib (Votrient^®^) and capsules of sunitinib (Sutent^®^) (Supplementary Material). The amount of P-CHO was 7-fold lower in commercial tablets than in the plasma of patients treated with pazopanib (37 ppm vs. median of 260 ppm, respectively). Conversely, the S-CHO level reached 210 ppm of the total amount of sunitinib extracted from commercial capsules, a value equivalent to the proportions measured in the microsomal (260 ppm) and cytosolic incubations (330 ppm).

## 4. Discussion

Identifying the causes and risk factors of pazopanib and sunitinib induced-hepatotoxicity is part of the challenge of improving these inhibitors’ management in metastatic renal cancer patients. However, understanding the mechanistic features resulting from liver damage is limited due to their complex and multifactorial origin [18]. After identifying pazopanib- and sunitinib-reactive aldehyde metabolites (P-CHO and S-CHO, respectively) [14], we evaluated their ability to induce hepatocyte cytotoxicity, mitochondrial dysfunction, and the accumulation of oxidative stress.

We observed that P-CHO exerted a cytotoxic effect at high concentrations (>50 µM) on the immortalized HepG2 cell line by activating apoptosis and impairing ATP synthesis, and we confirmed that pazopanib was not toxic in this case [13]. On the contrary, significant concentration-dependent toxicity and apoptosis activation were observed for sunitinib and its aldehyde. When using the HepaRG cell line that preserves more physiological characteristics, the function of human hepatocytes (especially phase I and phase II xenobiotic metabolism enzyme activities), and the expression of liver-specific genes [19], P-CHO toxicity was limited. S-CHO, however, affected cell viability to a greater extent than in HepG2 cells. These observations underlined the functional differences between the two cell lines in their ability to metabolize and eliminate drugs, as previously demonstrated by Paech et al. for sunitinib [20].

Important changes in mitochondria function and oxidative metabolism have been observed in cells treated with sunitinib. At high concentrations (>20 µM), sunitinib can inhibit both hepatic mitochondrial proliferation and respiration and reduce the glycolytic flux in HepG2 cells [12,20]. We completed these observations by showing that its aldehyde metabolite, S-CHO, decreases the maximal respiration rate in a concentration-dependent manner as it was demonstrated for its parent molecule, but it does not impact ATP production. Pazopanib-induced mitochondrial damages were less pronounced in a previous study [13], as it inhibited glycolysis only at high concentration (20 µM). Here, we showed that P-CHO impairs the ATP production in HepG2, especially when the cell relies on oxidative phosphorylation. Mitochondrial function or structure impairment can result in the increase of reactive oxygen species (ROS), such as mitochondrial superoxide, particularly when mitochondrial complexes III and I are inhibited [21]. Glutathione (GSH) is of paramount importance in protecting cells from accumulating ROS, and depletion is often associated with increased toxicity [22]. In our study, depleting GSH did not increase the accumulation of mitochondrial superoxide in cells exposed to aldehydes. Additionally, aldehydes did not significantly affect the expression of superoxide dismutases 1 and 2, two key regulators of ROS accumulation described as cytoplasmic and mitochondrial stress biomarkers [23]. Overall, both metabolites affect mitochondria in a different way that may reflect the effect of parent molecules on cell function.

In our previous study, we demonstrated that P-CHO and S-CHO can covalently bind the lysine residues of human proteins such as aldehyde-spiked albumin and plasma [6] when generated through biomimetic oxidations catalyzed by metalloporphyrin, HLM, and rCYP3A4. In this study, using a proteomic approach, we identified NADPH-cytochrome P450 reductase (POR) as a target of P-CHO. Crystal structure studies are needed to specifically identify local interactions between POR critical domains and P-CHO. This enzyme is essential in transferring electrons from NADPH, but large adducts located in the flavin cofactor domains may interfere with this critical step of the CYP450 reaction cycle [24]. POR has been demonstrated to generate ROS spontaneously, such as hydrogen peroxide (H_2_O_2_) and mitochondrial superoxide, as a result of molecular oxygen reduction by its flavin cofactors [25]. We observed a weak accumulation of superoxide at high concentrations (>100 µM) of P-CHO, meaning that the limited accumulation of ROS in HepG2 cells could result from POR inhibition by P-CHO adduct formation. Gray et al. [26] previously observed a similar effect of POR inhibition with 2-chloroethyl ethyl sulfide: spontaneous formation of ROS was NADPH-dependent and concomitant with the inhibition of POR-mediated substrate reduction.

The high level of reactivity of aldehydes towards proteins probably limits both the amounts detectable in the biological matrix and their clinical assessment. However, Wang et al. [27] detected P-CHO in the urine and feces (0.2% and <0.1%, respectively) of mice treated with pazopanib (300 mg/kg). Co-treatment with aminobenzotriazole (100 mg/kg) decreased these levels, confirming the role of CYP450 in aldehyde production. They also observed that aldehyde abundance in the urine correlated significantly with serum transaminase activities (r_ALT_ = 0.76, r_AST_ = 0.75, *p* < 0.05). Similarly, we evaluated P-CHO accumulation in the plasma of patients treated with pazopanib, but we observed no correlation with reported hepatic side-effects. Indeed, we detected only a small amount of P-CHO in these samples (around 10 ng/mL, i.e., 22 nM for P-CHO), and the cohort was too small to identify a concentration–response relationship. The concentrations of aldehyde used in vitro were thus very high (>2000 times) compared to those observed in plasma, constituting a limitation in our observations. However, the high reactivity of such metabolites suggests that their circulating concentration in plasma may not reflect the intra-cellular amount and that the possibility of monitoring plasma aldehydes seems of limited interest. Given the known hepatotoxicity of these drugs and despite the difference of metabolites levels between in vitro and in vivo conditions, we cannot exclude that the dysfunctions observed in vitro may not occur at a clinical level.

Sunitinib biotransformation to oxidized products through CYP450 metabolism has been widely described in vitro, in vivo, and in humans [28,29]. However, other oxidation pathways may be considered, especially when involving highly reactive species. Indeed, the accumulation of superoxides following high concentrations of sunitinib may trigger spontaneous formation of H_2_O_2_ that is then further reduced in reactive hydroxyl radicals, thereby, *in fine,* possibly oxidizing sunitinib. This mechanism has been previously reported for adriamycin auto-oxidation in liver microsomes [30]. Nevertheless, our attempts to determine the origin of S-CHO and P-CHO led us to consider that a non-biological process could also contribute to aldehyde formation, as shown by its significant presence in sunitinib capsules, along with the impossibility of generating it with microsomes. Notably, Patel et al. [31] identified P-CHO as a photolytic degradation product of pazopanib. Although we detected extremely low levels of aldehyde (between 37 ppm and 330 ppm), they were above the 20 ppm limit imposed by the European Pharmacopoeia for an impurity of Imatinib mesylate, a precursor of a reactive quinone imine (4-methyl-N3-[4-(pyridin-3-yl)pyrimidin-2-yl] benzene-1,3-diamine) [32].

Finally, growing evidence suggests that reactive derivatives may activate an immune response and inflammation processes based on two major and complementary hypotheses. A reactive metabolite can act as a hapten to produce neoantigens (the hapten hypothesis), and the accumulation of these immunogenic molecules can cause cell damage followed by the release of danger-associated molecular patterns (DAMPs), activating antigen-presenting cells (APCs, the danger hypothesis) [33]. Kato et al. [34] tested the ability of gefitinib hepatic reactive metabolites to induce DAMP releases from monocytes. They showed that these metabolites could activate inflammasomes in APCs, a mechanism that could be similarly triggered by pazopanib or sunitinib.

To conclude, this study aimed to elucidate the contribution of pazopanib and sunitinib aldehyde derivatives to the mechanisms of TKI liver toxicity. Although electrophile aldehyde moiety is known to confer high levels of reactivity, the effects produced by P-CHO and S-CHO are slightly different, but both alter the hepatocyte’s function. Our findings demonstrated that the former may be responsible for a moderate but observable effect on the mitochondrial ability to produce ATP in HepG2 cells. A specific interaction of P-CHO was observed with NADPH-cytochrome P450 reductase (POR), whose consequence on CYP450 function should be further addressed. Moreover, hepatotoxicity is a complex issue depending on multiple mechanisms and interactions between biological pathways that need to be further understood.

## Data Availability

All datas are in the Supplementary Materials and are available on request from the corresponding author.

## Supplementary Materials

The following supporting information can be downloaded at: https://www.mdpi.com/article/10.3390/metabo12090852/s1. Figure S1: cytotoxicity of sunitinib, pazopanib and respective aldehydes after pre-treatment with BSO (250 µM). Figure S2: Proteomic analyses of microsomes incubated with pazopanib aldehyde (P-CHO). Figure S3: The MS/MS fragmentation spectrum of the EELAQFHRDGALTQLNVAFSR peptide (NADPH-cytochrome P450 reductase, Uniprot P16435) obtained after microsomes incubation with pazopanib aldehyde. Figure S4: metabolic pathways of production of P-CHO and S-CHO. Table S1: Variable modifications and their respective mass shifts, used for microsomes peptide identification.
