# Supplementary material for: Biological Role of Pazopanib and Sunitinib Aldehyde Derivatives in Drug-Induced Liver Injury
Source: Metabolites. 2022 Sep 11;12(9):852. doi: 10.3390/metabo12090852 (PMC9505977; doi:10.3390/metabo12090852)
Supplement: Supplementary file 1 [file metabolites-12-00852-s001.zip › metabolites-1890039-supplementary.pdf]

Biological role of pazopanib and sunitinib aldehyde derivatives in drug-induced liver injury

Supplementary Methods

Proteomic analysis: mass spectrometry

Microsomes digested peptides were solubilized in 10% acetonitrile 0.05% TFA and analyzed on a SCIEX 5600+ TripleTOF mass spectrometer operated in DDA mode. A Dionex Ultimate 3000 nanoLC HPLC system and a Hypersil GOLD 150x0.32 mm column (Thermo Scientific), packed with C18, 3 µm, and 175 Å material, were used for peptide separation. For the HPLC method, buffer A was 0.1% (v/v) formic acid, and buffer B was 0.1% (v/v) formic acid, 90% (v/v) acetonitrile. The gradient was 4-7% of buffer B in 3 min, 7–45% of buffer B in 41 min, 45-95% of buffer B in 2min, 95% plateau of buffer B for 4 min, 95-4% of buffer B in 1 min with a flow rate of 5 µL/min. For the MS method, a survey scan at the MS1 level (350–1600 m/z) was first carried out with 250 ms per scan. The Top 20 most intense precursors, with charge states of 2–4, were then fragmented. Signals exceeding 75 counts per second were selected for fragmentation and MS2 spectra generation. MS2 spectra were collected in the mass range 100–1600 m/z for 80 ms per scan. The dynamic exclusion time was set at 10 s.

Proteomic analysis: LC-MS data analysis

To identify pazopanib modified peptides, profile-mode.wiff files from data acquisition were centroided and converted to mzML and MGF format using MSConvert v3.0.2 and submitted to MSFragger[1] v3.1.1 + FragPipe v14.0 and SearchGUI[2] v4.0.39 + PeptideShaker 2.0.33[3], with simultaneous selection of X!tandem, MSGF+, OMSSA, Tide and COMET search engines. MS/MS spectra were compared with the UniProt[4] Swiss-Prot human database. Trypsin was chosen as the enzyme, and two missed cleavages were allowed. Peptide tolerance at MS and MS/MS level were set at 30 ppm and 0.2 Da, respectively with C carbamidomethylation as a/the fixed modification. Peptide variable modifications allowed during the analysis are shown in Supplementary Table 1. To limit the number of simultaneous modifications to be computed, lysine modifications and cysteine + histidine were performed in two independent searches.

Liver microsomal and cytosolic incubations

For microsomal incubations, a mixture containing 2 mg/mL of pooled human liver microsomes (ref. #452161, Corning, Bedford, US), NADPH regenerating solutions A (20X, 12.5 µL/250 µL, ref. #451220, Corning) and B (100X, 2.5 µL/250 µL, ref. #451200, Corning) was prepared in a warm phosphate buffer (0.1M, pH = 7.4, 37°C). The final volume was 250 µL after adding sunitinib and pazopanib at a concentration of 20 µM (total amount of DMSO <0.2%). Negative control was performed without NADPH. The reaction was stopped after 90 minutes at 37°C by adding 250 µL of acetonitrile to

[1] Kong, Andy T., et al. "MSFragger: ultrafast and comprehensive peptide identification in mass spectrometry–based proteomics." *Nature methods* 14.5 (2017): 513-520.

[2] Barsnes H and Vaudel M: SearchGUI: a highly adaptable common interface for proteomics search and de novo engines. J Proteome Res. 2018;17(7):2552-2555.

[3] Vaudel et al. Nature Biotechnol. 2015 Jan;33(1):22–24.

[4] UNIPROT CONSORTIUM. UniProt: the universal protein knowledgebase. *Nucleic acids research*, 2016, vol. 45, no D1, p. D158-D169.

precipitate proteins. The mixture was centrifuged at 10,000 g for 15 minutes. The supernatant was filtered with a tuberculin 2.0 mL syringe and a Nylon Acrodisc® Syringe filter (0.2µM, 13 mm, Waters, Milford, MA) prior to analysis by UPLC-MS/MS. A similar protocol was conducted with human liver cytosol (Corning® UltraPool™ cytosol human liver 150 donors pool, ref. #452115). Extracted samples were analyzed by UPLC/MS-MS, as described in the main text.

Analyses of Votrient® and Sutent® commercial drugs
P-CHO and S-CHO amounts were measured in the pharmaceutical forms of pazopanib (Votrient®, Novartis) and sunitinib (Sutent®, Pfizer), respectively. Five tablets of 200 mg of Votrient® were crushed into fine powder, and an equivalent of 2.5 mg of pazopanib was completely dissolved in DMSO to obtain a solution of 1 mg/mL. For sunitinib, the entire contents of a 25 mg capsule of Sutent® was dissolved in 5 mg/mL in DMSO and then diluted to a concentration of 1 mg/mL. The solutions were filtered through a 0.22 µm membrane and diluted to either 30,000 ng/mL or 10,000 ng/mL in ACN. Similar samples were prepared in plasma and extracted by ACN precipitation, as described in the previous section. Extracted samples were analyzed by UPLC/MS-MS, as described in the main text. The ratio of areas (aldehyde:parent molecule) was used to express a relative concentration (%) of the metabolite.

Supplementary Results

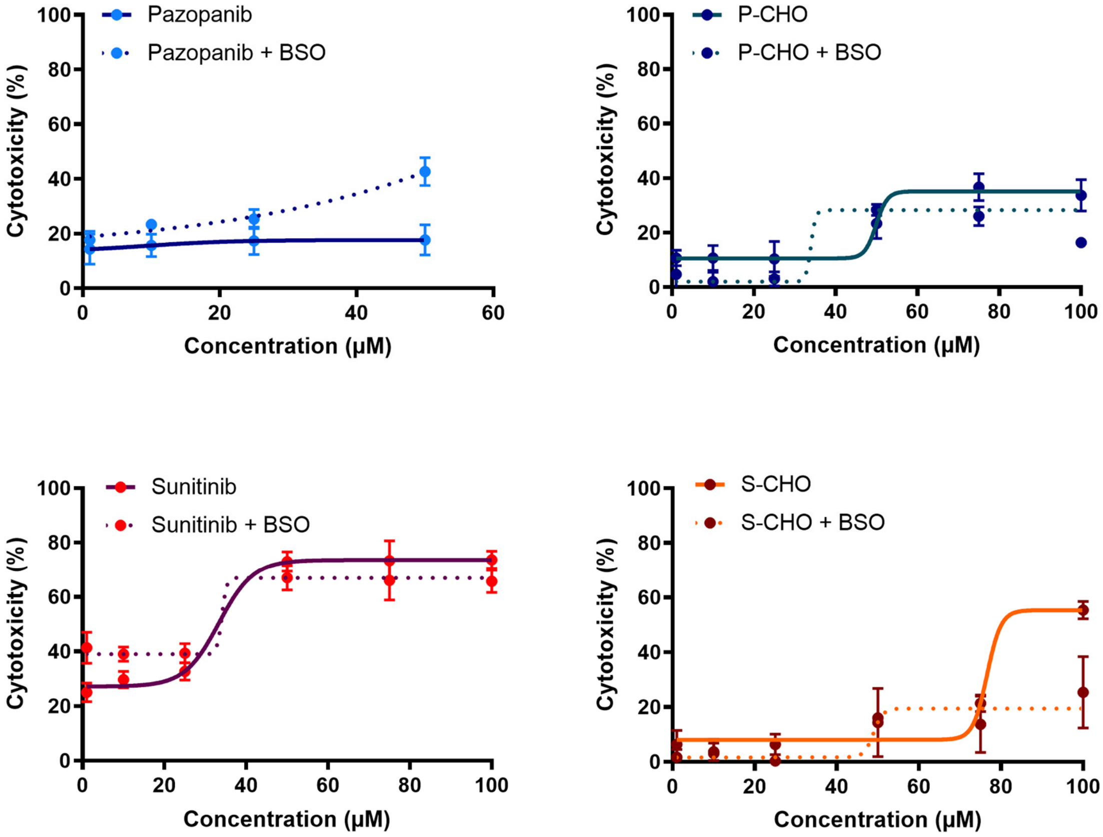

Supplementary Figure S1: cytotoxicity of sunitinib, pazopanib and respective aldehydes after pre-treatment with BSO (250 µM). Glutathione was expected to decrease under these conditions and to induce accumulation of reactive species in response to the presence of aldehydes. No significant difference was observed between the two conditions. Results are the mean + s.d. of three independent evaluations. BSO: buthionine sulfoximine; P-CHO: pazopanib aldehyde; S-CHO: sunitinib aldehyde

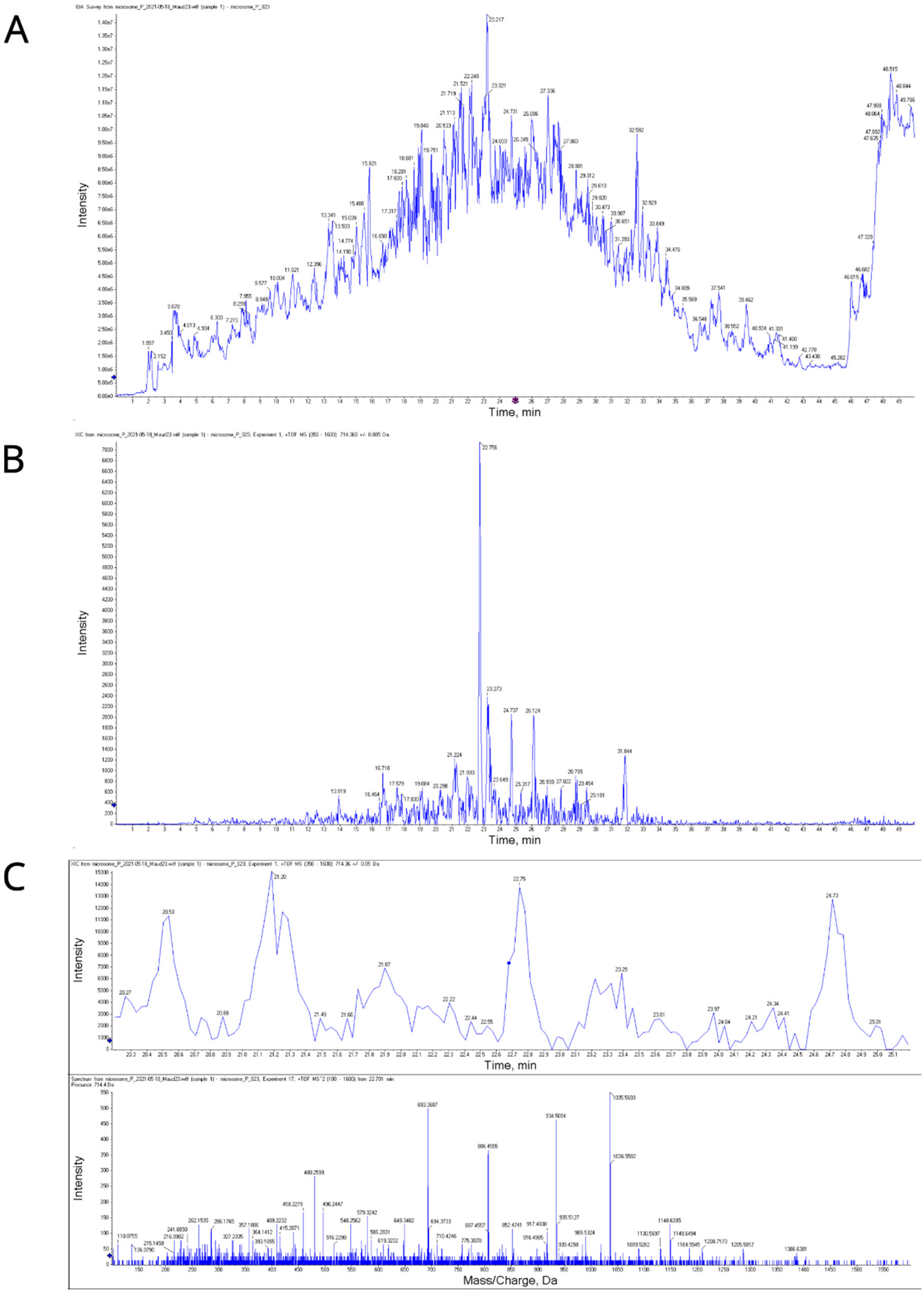

Supplementary Figure S2: Proteomic analyses of microsomes incubated with pazopanib aldehyde (P-CHO).
A : Total Ion Chromatogram (TIC) of the tryptic digest of the microsomes incubated with (P-CHO).
B : Extracted Ion Chromatogram (XIC) of the m/z 714.36 corresponding to the peptide modified by P-CHO
C: top panel : zoom of the XIC of the m/z 714.36 corresponding to the peptide modified by P-CHO
bottom panel : MSMS spectrum of the peptide EELAQFHRDGALTQLNVAFSR modified by P-CHO.

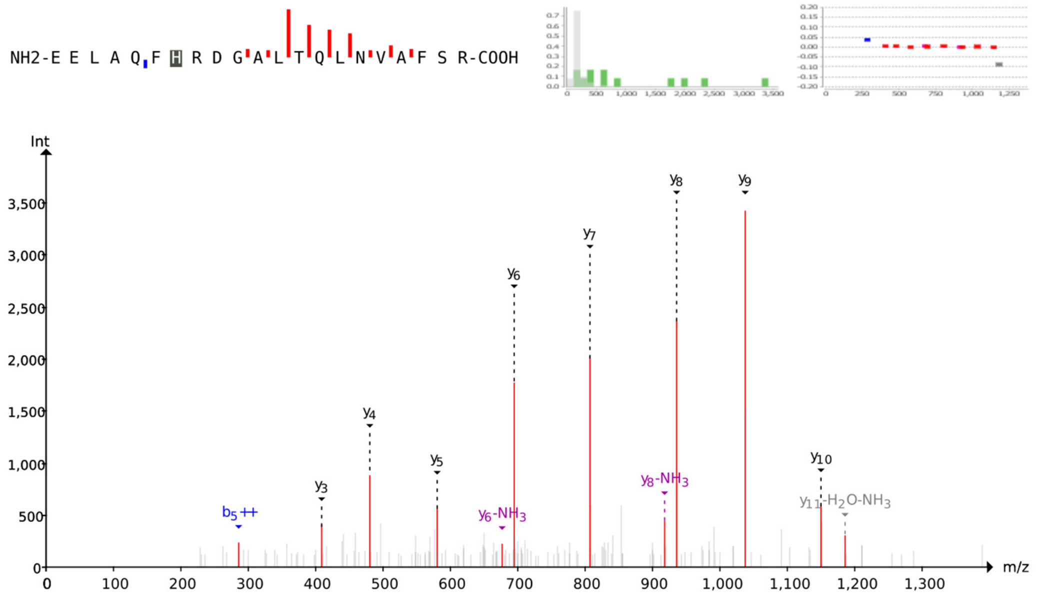

Supplementary Figure S3: The MS/MS fragmentation spectrum of the EELAQFHRDGALTQLNVAFSR peptide (NADPH-cytochrome P450 reductase, Uniprot P16435) obtained after microsomes incubation with pazopanib aldehyde. This peptide was found to be modified by pazopanib aldehyde on histidine 7. The histidine modification was validated by three different databases for peptide identification: MSFragger, OMSSA and Comet. Y ions are in red and B ions in blue. Mass accuracy of the Y, B fragments was < 0.05 ppm (top right).

Supplementary Table S1: Variable modifications and their respective mass shifts, used for microsomes peptide identification.

**Peptide Sequence**	EELAQFHRDGALTQLNVAFSR
**Modified Sequence**	EELAQF**H[588]**RDGALTQLNVAFSR
**Peptide Length**	21
**M/Z**	714,0963
**Charge**	4
**Observed Mass**	2852,3562
**Probability**	1
**Expectation**	0,0004
**Spectral Count**	1
**Intensity**	0
**Assigned Modifications**	7H(451.1427)
**Protein ID**	P16435
**Entry Name**	NCPR_HUMAN
**Gene**	POR
**Protein Description**	NADPH-cytochrome P450 reductase

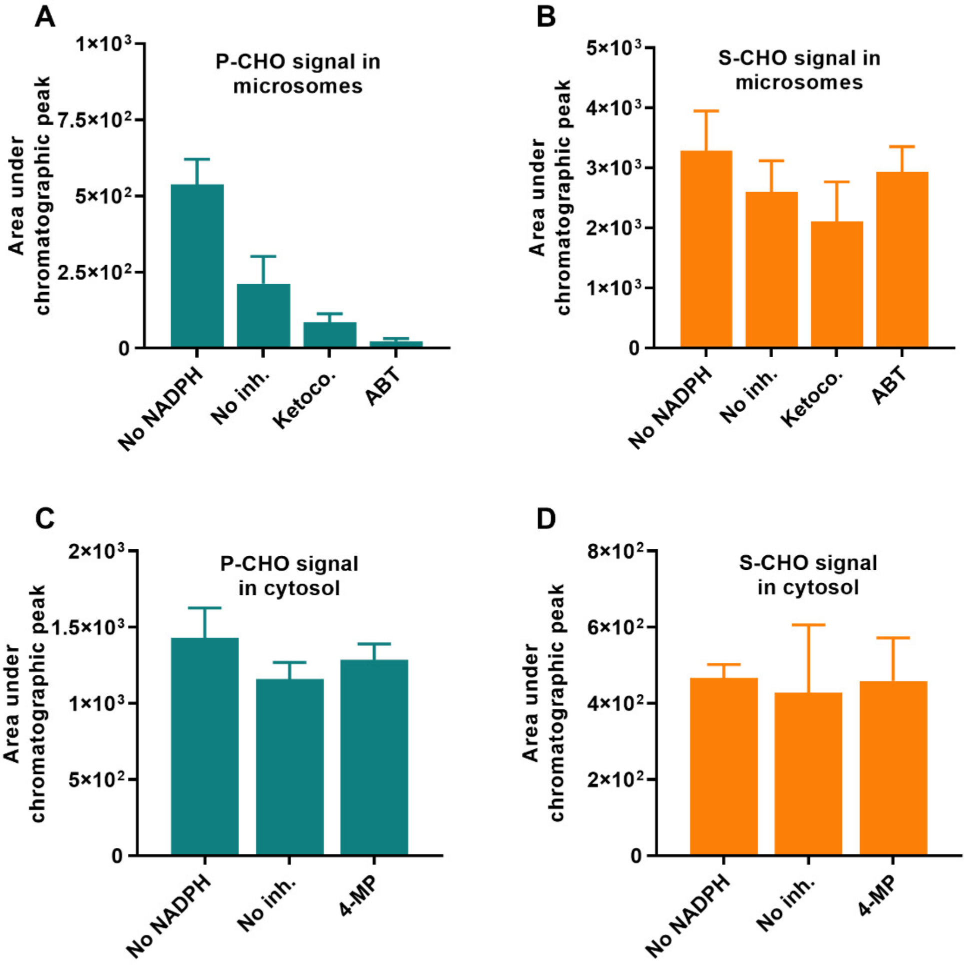

Supplementary Figure S4: metabolic pathways of production of P-CHO and S-CHO. Pazopanib (S4A and S4C) and sunitinib (S4B and S4D) (20 µM) were first incubated with liver microsomes with or without CYP450 inhibitors (ketoconazole 50 µM, to inhibit CYP3A4 or aminobenzotriazole, ABT, 500 µM, a pan-CYP450 inhibitor). A second incubation with hepatocyte cytosolic fractions in presence of alcohol dehydrogenase inhibitor 4-methylpyrazole (4-MP, 500 µM, in cytosol fraction) was performed. The aldehyde derivatives P-CHO (S4A and S4C) and S-CHO (S4B and S4D) were detected by UPLC-MS/MS and the signal was compared to the control condition (no NADPH regeneration system). Data represents the mean ± s.d. of three independent experiments. * *P* value < 0.05 for the comparison with negative control (No NADPH).

Only traces of P-CHO were detected in the incubations and the signal was at its highest level in absence of NADPH ("No NADPH", inactive microsomes). The amount of P-CHO was reduced and was almost undetectable in the presence of ABT (Figure S3A). The highest signal of S-CHO was found under control incubation conditions, and equivalent levels were detected whether in presence or absence of inhibitors. Of note, other known metabolites such as alcohols and N-desethyl-sunitinib were quantifiable in each experiment and decreased in the presence of CYP inhibitors, attesting for the good functioning of the microsomes system (data not shown).
